# Updated results from GEST study: a randomized, three-arm phase III study for advanced pancreatic cancer

**DOI:** 10.1007/s00432-017-2349-y

**Published:** 2017-02-16

**Authors:** Takuji Okusaka, H. Miyakawa, H. Fujii, S. Nakamori, T. Satoh, Y. Hamamoto, T. Ito, H. Maguchi, S. Matsumoto, H. Ueno, T. Ioka, N. Boku, S. Egawa, T. Hatori, J. Furuse, K. Mizumoto, S. Ohkawa, T. Yamaguchi, K. Yamao, A. Funakoshi, J. S. Chen, A. L. Cheng, A. Sato, Y. Ohashi, M. Tanaka, on behalf of the GEST group

**Affiliations:** 1 Department of Hepatobiliary and Pancreatic Oncology, National Cancer Center Hospital, 5-1-1 Tsukiji, Chuo-ku, Tokyo, 104-0045 Japan; 2 Division of Biliopancreatology, Sapporo Kosei General Hospital, Sapporo, Japan; 3 Division of Clinical Oncology, Jichi Medical University, Tochigi, Japan; 4 Hepato-Biliary-Pancreatic Surgery, Osaka National Hospital, Osaka, Japan; 5 Department of Frontier Science for Cancer and Chemotherapy, Osaka University Graduate School of Medicine, Suita, Japan; 6 Keio Cancer Center, Keio University Hospital, Tokyo, Japan; 7 Department of Medicine and Bioreguratory Science, Graduate School of Medical Sciences, Kyushu University, Fukuoka, Japan; 8 Center for Gastroenterology, Teine-Keijinkai Hospital, Sapporo, Japan; 9 Department of Medical Oncology, Kyoto University Hospital, Kyoto, Japan; 10 Department of Hepatobiliary and Pancreatic Oncology, Osaka Medical Center for Cancer and Cardiovascular Diseases, Osaka, Japan; 11 Gastrointestinal Medical Oncology Division, National Cancer Center Hospital, Tokyo, Japan; 12 Department of Surgery, Tohoku University, Sendai, Japan; 13 Department of Surgery and Digestive Diseases Center, International University of Health and Welfare Mita Hospital, Tokyo, Japan; 14 Department of Medical Oncology, Kyorin University School of Medicine, Tokyo, Japan; 15 Kyushu University Hospital Cancer Center, Fukuoka, Japan; 16 Division of Hepatobiliary and Pancreatic Oncology, Kanagawa Cancer Center, Yokohama, Japan; 17 Department of Gastroenterology, Chiba Cancer Center, Chiba, Japan; 18 Department of Gastroenterology, Aichi Cancer Center Hospital, Nagoya, Japan; 19 Division of Pancreatology, Fukuoka Sanno Hospital, Fukuoka, Japan; 20 Division of Hematology-Oncology, Linkou Chang Gung Memorial Hospital and Chang Gung University, Tao-Yuan, Taiwan, Republic of China; 21 Department of Oncology, National Taiwan University Hospital, and National Taiwan University Cancer Center, Taipei, Taiwan, Republic of China; 22 Department of Medical Oncology, Hirosaki University Graduate School of Medicine, Aomori, Japan; 23 Department of Integrated Science and Engineering for Sustainable Society, Chuo University, Tokyo, Japan; 24 Department of Surgery, Shimonoseki City Hospital, Shimonoseki, Japan

**Keywords:** Pancreatic cancer, Gemcitabine, S-1, Updated data, Subgroup analysis

## Abstract

**Purpose:**

The GEST study showed non-inferiority of S-1 but not superiority of gemcitabine plus S-1 (GS) to gemcitabine alone for overall survival with the data by the cut-off date of 31st July in 2010 for chemo-naïve patients with advanced pancreatic cancer. We considered it important to determine whether S-1 maintains non-inferiority after a long-term follow-up in the GEST study and to obtain a firm positive conclusion. In addition, it may be an interesting challenge to explore the efficacious profile of GS in the long-term follow-up study. Using the data from the follow-up period, background and efficacy in patients from Taiwan and Japan, as well as the rates of tumor shrinkage in locally advanced and metastatic patients (Waterfall plot) were also analyzed.

**Methods:**

The results of the primary analysis were reconfirmed, and subset analysis of overall survival and progression-free survival was performed based on the overall survival data updated by the cut-off date of 31st July in 2011.

**Results:**

The median follow-up period was 29.8 months, and 795 deaths occurred (95.6%). The median overall survival was 8.8 months for gemcitabine, 9.7 months for S-1 (hazard ratio [HR], 0.96; 97.5% confidence interval [CI], 0.79–1.17), and 9.9 months for GS (HR 0.91; 97.5% CI 0.75–1.11). In patients with performance status (PS) 0, the median overall survival was 9.8 months for gemcitabine, 10.9 months for S-1, and 10.5 months for GS. In patients with PS 1, the median overall survival was 6.2 months for gemcitabine, 6.3 months for S-1, and 9.6 months for GS.

**Conclusion:**

Our survey reconfirmed the non-inferiority of S-1 to gemcitabine and showed S-1 can be used as one of the standard treatment options for advanced pancreatic cancer.

**Trial registration:**

ClinicalTrials.gov: NCT00498225.

**Electronic supplementary material:**

The online version of this article (doi:10.1007/s00432-017-2349-y) contains supplementary material, which is available to authorized users.

## Introduction

In various clinical trials, gemcitabine has been adapted as a standard treatment in patients with advanced unresectable pancreatic cancer (Burris et al. 1997). Recently, it has been shown that gemcitabine plus erlotinib (Moore et al. 2007), a combination of 5-fluorouracil, leucovorin, irinotecan, and oxaliplatin (FOLFIRINOX) (Conroy et al. 2011), and gemcitabine plus albumin-bound paclitaxel (nab-paclitaxel) (Von Hoff et al. 2013) are superior to gemcitabine alone. Concerns have been raised about the risk of adverse events of these combination chemotherapy regimens, because medical condition is not so good in patients with advanced pancreatic cancer. There are still unmet needs in the first-line chemotherapy especially for its feasibility.

An oral fluoropyrimidine, S-1 (TS-1; a combination of tegafur, gimeracil, and oteracil; Taiho Pharmaceutical Co. Ltd., Tokyo, Japan, http://www.taiho.co.jp/) is also used for treatment of pancreatic cancer in Japan. Starting in 2007, we performed a randomized three-arm phase III study to evaluate the non-inferiority of S-1 monotherapy and superiority of gemcitabine plus S-1 (GS) to gemcitabine in patients with locally advanced and metastatic pancreatic cancer (GEST study). The results demonstrated that S-1 monotherapy was non-inferior to gemcitabine in terms of overall survival (OS) (Ueno et al. 2013). While OS in the GS group was not significantly better than that in the gemcitabine, a survival benefit was suggested in the subgroups of patients with locally advanced disease and those with performance status (PS) 1.

Recently, FOLFIRINOX and gemcitabine plus nab-paclitaxel, compared with gemcitabine alone, showed significantly prolonged survival; however, both regimens had increased toxicity. Monotherapy with gemcitabine or S-1, associated with mild adverse events, is still recognized as a standard treatment option in the guideline from Japan Pancreas Society. We considered it important to determine whether S-1 maintains non-inferiority after a long-term follow-up in the GEST study and to obtain a firm positive conclusion. In addition, it may be an interesting challenge to explore the efficacious profile of GS in the long-term follow-up study.

In the previous analysis, OS was estimated on the basis of 710 deaths (85.3%) among the 832 patients (median follow-up, 18.4 months). In the present study, we followed-up the survivors and updated the OS data to obtain more robust conclusions. We now report the long-term outcomes of the GEST study and the detail results of the additional analysis.

## Patients and methods

The details of the patient characteristics and study methods were reported previously (Ueno et al. 2013).

### Patients

The main eligibility criteria were as follows: locally advanced or metastatic pancreatic cancer; histologically or cytologically confirmed diagnosis of adenocarcinoma; no prior chemotherapy or radiotherapy for pancreatic cancer; age of 20–80 years; Eastern Cooperative Oncology Group PS of 0–1; and adequate main organ function. Written informed consent was obtained from all eligible patients.

### Study design and treatment

This phase III study was a multicenter, open-label, randomized controlled trial, sponsored by Taiho Pharmaceutical Co. Ltd. in Japan and TTY Biopharm Co. Ltd. in Taiwan. The study was performed as a post-marketing study in Japan and a registration study in Taiwan, and was in compliance with the Declaration of Helsinki. The study was registered with ClinicalTrials.gov (NCT00498225) and approved by the institutional review board or ethics committee of each participating center.

The patients were randomly assigned to one of three treatment groups in a 1:1:1 ratio with minimization method stratifying by extent of disease (locally advanced vs. metastatic) and institution. The patients assigned to gemcitabine alone were given gemcitabine (1000 mg/m^2^) as an intravenous infusion over the course of 30 min on days 1, 8, and 15 in a 28-day cycle. The patients assigned to S-1 alone were given S-1 orally twice-daily at a dose based on their body-surface area (<1.25 m^2^, 80 mg/day; ≥1.25 to <1.5 m^2^, 100 mg/day; ≥1.5 m^2^, 120 mg/day) on days 1–28 in a 42-day cycle. The patients assigned to GS received gemcitabine (1000 mg/m^2^) as an intravenous infusion on days 1 and 8 plus S-1 orally twice-daily at a dose based on their body-surface area (<1.25 m^2^, 60 mg/day; ≥1.25 to <1.5 m^2^, 80 mg/day; ≥1.5 m^2^, 100 mg/day) on days 1–14 in a 21-day cycle.

### Assessments

OS was calculated on the basis of follow-up data available at the cut-off point of 31 June 2011. Because other data were not updated by follow-up, progression-free survival (PFS) was calculated on the basis of the previous main analysis at the cut-off point of 31 July 2010 (Ueno et al. 2013). Objective tumor response was assessed according to the Response Evaluation Criteria in Solid Tumors (RECIST), version 1.1. We further performed exploratory subgroup analyses of OS and PFS not pre-specified in the protocol according to PS and disease extension, and separately assessed long-term survival in Japanese and Taiwanese patients.

### Statistical analysis

Data for patients included in the full analysis set (FAS) were analyzed. The FAS population was composed of all randomized subjects with written informed consent. The Kaplan–Meier method was used to estimate the distribution of events over time in each treatment group. The 95% confidence intervals (CIs) for median survival time were calculated using the method of Brookmeyer and Crowley (1982). The 95% CIs for cumulative survival rates were calculated using Greenwood’s formula (Kalbfleisch et al. 1980). Hazard ratios (HRs) were estimated by a Cox-proportional hazards model, stratified by country (Japan vs. Taiwan) and extent of disease (locally advanced vs. metastatic). For HRs, 97.5% CIs were calculated, taking multiplicity into account.

In the subgroup analysis, interaction test was performed to evaluate heterogeneity in treatment effects among the subgroups. All *P* values were two-sided. Changes in tumor size were calculated as the percentage changes from baseline to nadir. Data analyses were performed with SAS, version 9.1.3 (SAS Institute, Cary, NC, USA).

## Results

### Patients

Between July 2007 and October 2009, a total of 834 patients were enrolled from 75 institutions in Japan and Taiwan (768 in Japan and 66 in Taiwan). In the GS group, two patients without written informed consent were excluded from the study. The FAS thus comprised 832 patients (Supplemental Fig. 1). The patients’ background characteristics were well balanced among the three treatment groups. In the previous report (Ueno et al. 2013), the analysis of OS was based on 710 deaths, and the remaining 122 patients were followed-up for this updated analysis. At the completion of follow-up, 795 events were observed (95.6%). As additional information, the characteristics are separately presented for patients in Japan and Taiwan (Table 1). The major differences in the patient background characteristics between Japan and Taiwan were age (<65/≥65), PS (0/1), extent of disease (locally advanced/metastatic), and tumor location (head/body/tail).

**Table 1:** Baseline characteristics by country

Characteristic	Japan (*n* = 766)		Taiwan (*n* = 66)		*P* (χ^2^ test)
	No.	%	No.	%	
Sex					
Male	458	59.8	40	60.6	0.9
Female	308	40.2	26	39.4	
Age (years)					
<65	368	48.0	48	72.7	<0.001
≥65	398	52.0	18	27.3	
ECOG PS					
0	503	65.7	28	42.4	<0.001
1	263	34.3	38	57.6	
Extent of disease					
Locally advanced	193	25.2	9	13.6	0.04
Metastatic	573	74.8	57	86.4	
Type of tumor					
Adenocarcinoma	754	98.4	66	100	0.31
Adenosquamous carcinoma	12	1.6	0	0	
Pancreas excision					
No	708	92.4	58	87.9	0.19
Yes	58	7.6	8	12.1	
Tumor location^a^					
Head	326	45.6	22	33.3	0.14
Body	291	38.0	23	34.8	0.61
Tail	162	21.1	27	40.9	<0.001
Biliary drainage					
No	573	74.8	55	83.3	0.12
Yes	193	25.2	11	16.7	

*ECOG PS* Eastern Cooperative Oncology Group Performance Status

^a^Including patients with tumors involving multiple sites

### Efficacy

**Fig. 1:**
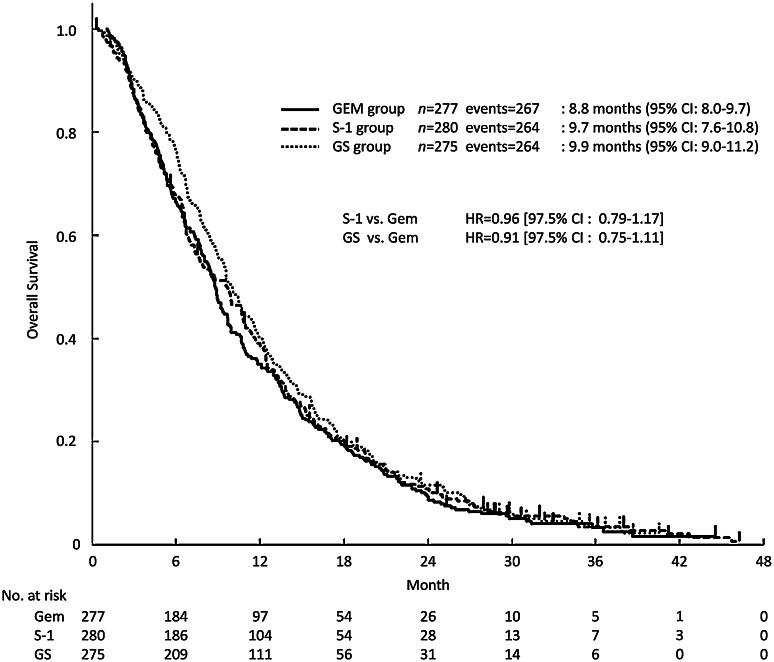
Kaplan–Meier curves for updated OS in the full analysis set. *CI* confidence interval, *GEM* gemcitabine, *GS* gemcitabine plus S-1, *HR* hazard ratio, *OS* overall survival

The median follow-up period was 29.8 months (range 0.3–46.3). The median OS (mOS) was 8.8 months (95% CI 8.0–9.7) in the gemcitabine group, 9.7 months (95% CI 7.6–10.8) in the S-1 group (HR 0.96; 97.5% CI 0.79–1.17), and 9.9 months (95% CI 9.0–11.2) in the GS group (HR 0.91; 97.5% CI 0.75–1.11) (Fig. 1). The survival rates at 1, 2, and 3 years were 35.0, 9.4, and 3.4% in the gemcitabine group, 38.4, 10.9, and 3.6% in the S-1 group, and 40.4, 11.6, and 4.1% in the GS group (Supplemental Table 1).

The median tumor shrinkage ratio, calculated using the sum of the longest diameter of target lesions at baseline and its nadir, was 7.0, 7.9, and 20.9% for pancreatic primary lesions, and 3.6, 10.4, and 18.8% for metastatic lesions in the gemcitabine group, S-1 group, and GS group, respectively (Supplemental Table 2 and Supplemental Fig. 2a–d).

### Subgroup analyses

**Fig. 2:**
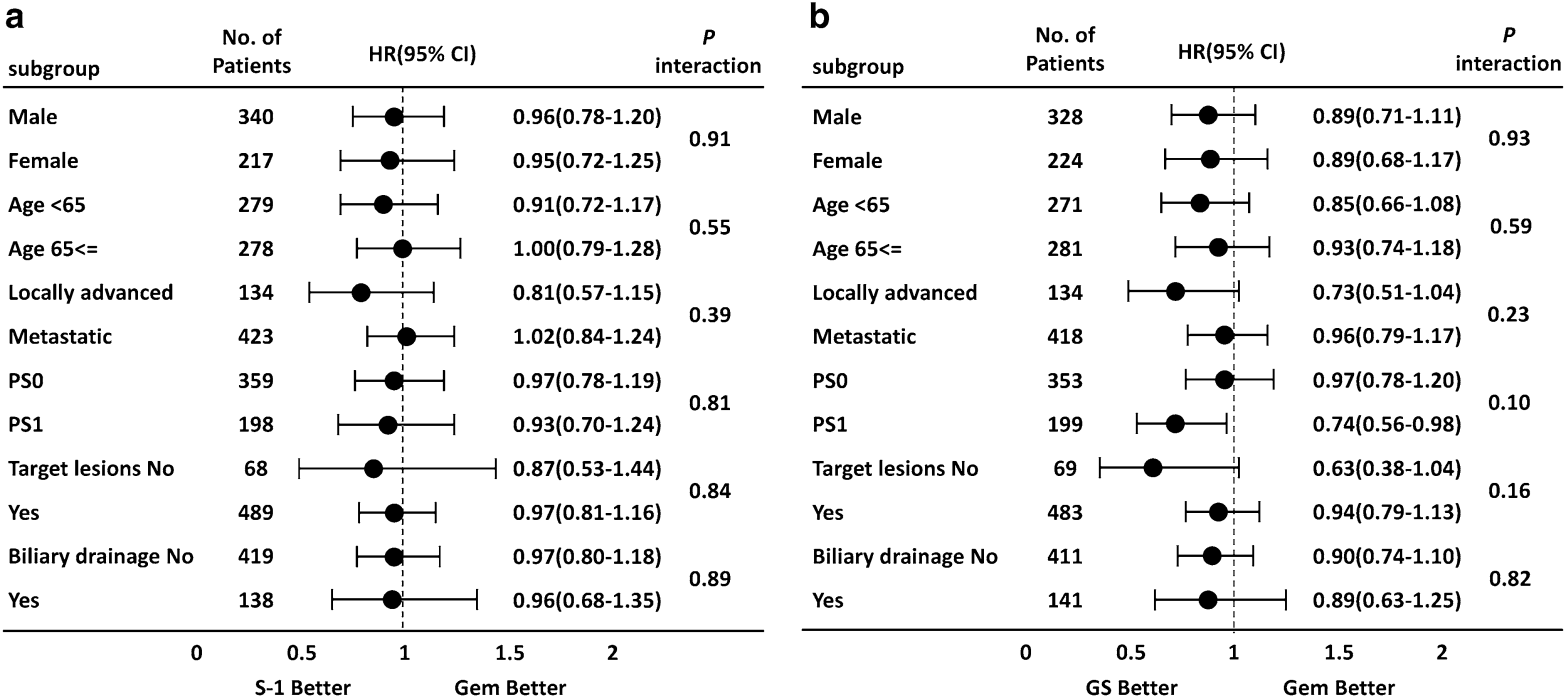
Forest plots of treatment effects on OS in subgroup analyses. **a** S-1 vs. gemcitabine. **b** GS vs. gemcitabine. *CI* confidence interval, *GEM* gemcitabine, *GS* gemcitabine plus S-1, *HR* hazard ratio, *OS* overall survival, *PS* performance status

Results of a subgroup analysis at the primary analysis have already been reported (Ueno et al. 2013), and similar results were confirmed in this long-term follow-up study. Comparing S-1 and gemcitabine, there were no significant interactions in any of the subgroups (Fig. 2a). In addition, there was no significant interactions that were observed in any subgroups comparing between GS and gemcitabine. However, as reported in the primary analysis, there was a trend toward the GS group demonstrating better OS than the gemcitabine group in patients with a PS of 1 and those with locally advanced disease (Fig. 2b). While the hazard ratios of the GS group vs. the gemcitabine group were 0.69 (95% CI 0.51–0.92) in patients with a PS of 1 and 0.67 (95% CI 0.46–0.99) in patients with locally advanced cancer in the report of the primary analysis, and the ratios were 0.74 (95% CI 0.56–0.98) in patients with a PS of 1 and 0.73 (95% CI 0.51–1.04) in patients with locally advanced cancer in the follow-up analysis.

**Fig. 3:**
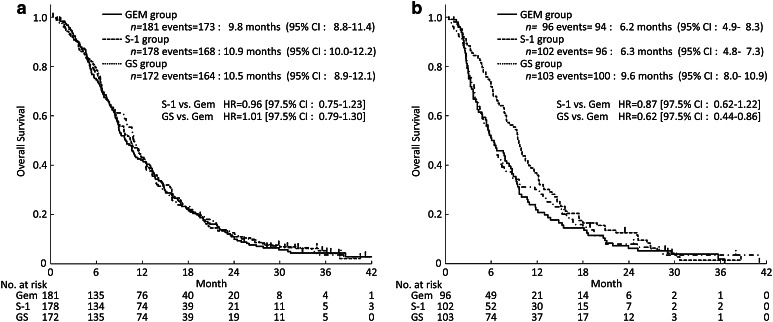
Kaplan–Meier plots for updated OS in the subset analysis (**a**, PS 0; **b**, PS 1). *CI* confidence interval, *GEM* gemcitabine, *GS* gemcitabine plus S-1, *HR* hazard ratio, *PS* performance status

The survival curves according to PS are shown in Fig. 3 In patients with PS 0, the mOS was 9.8 months (95% CI 8.8–11.4) in the gemcitabine group, 10.9 months (95% CI 10.0–12.2) in the S-1 group (HR 0.96; 97.5% CI 0.75–1.23), and 10.5 months (95% CI 8.9–12.1) in the GS group (HR 1.01; 97.5% CI 0.79–1.30) (Fig. 3a). In patients with PS 1, the mOS was 6.2 months (95% CI 4.9–8.3) in the gemcitabine group, 6.3 months (95% CI 4.8–7.3) in the S-1 group (HR 0.87; 97.5% CI 0.62–1.22), and 9.6 months (95% CI 8.0–10.9) in the GS group (HR 0.62; 97.5% CI 0.44–0.86) (Fig. 3b).

In patients with PS 0, the median PFS (mPFS) was 4.4 months (95% CI 3.5–5.4) in the gemcitabine group, 4.2 months (95% CI 3.4–4.7) in the S-1 group (HR 1.03; 97.5% CI 0.80–1.31), and 6.1 months (95% CI 5.5–7.2) in the GS group (HR 0.70; 97.5% CI 0.55–0.90). In patients with PS 1, the mPFS was 2.5 months (95% CI 1.8–4.1) in the gemcitabine group, 2.3 months (95% CI 1.6–3.8) in the S-1 group (HR 1.15; 97.5% CI 0.83–1.61), and 5.4 months (95% CI 4.2–6.8) in the GS group (HR 0.52; 97.5% CI 0.37–0.73).

The proportions of patients who received both drugs (gemcitabine and S-1) in their first-line and second-line treatments were 63.5% (115/181) in the gemcitabine group and 65.7% (117/178) in the S-1 group among patients with PS 0, and 44.8% (43/96) in the gemcitabine group and 55.9% (57/102) in the S-1 group among patients with PS 1.

In Japan, the updated mOS was 8.9 months (95% CI 8.3–9.7) in the gemcitabine group, 9.7 months (95% CI 7.7–10.9) in the S-1 group (HR 0.96; 97.5% CI 0.78–1.17), and 9.7 months (95% CI 8.9–10.9) in the GS group (HR 0.91; 97.5% CI 0.74–1.11). In Taiwan, the mOS was 5.3 months (95% CI 4.2–10.8) in the gemcitabine group, 6.4 months (95% CI 4.8–11.4) in the S-1 group (HR 1.07; 97.5% CI 0.50–2.27), and 11.2 months (95% CI 8.0–17.1) in the GS group (HR 0.96; 97.5% CI 0.45–2.05). The updated cumulative survival rates at 1, 2, and 3 years are shown according to country (Japan vs. Taiwan) and disease stage (locally advanced vs. metastatic) in Supplemental Table 1.

## Discussion

Our analysis of updated follow-up data for the GEST study reconfirmed that S-1 was non-inferior to gemcitabine, while GS was not shown to be superior to gemcitabine (Fig. 1). These findings were consistent with the results of the primary analysis. In comparisons of patients in Japan and Taiwan, the outcomes for the S-1 group and gemcitabine group were found to be slightly poorer in patients in Taiwan than in Japan. These differences were attributed to the fact that the proportions of patients with PS 1 and metastatic disease, which are generally associated with poorer outcomes, were slightly higher for patients in Taiwan than in Japan (Table 1).

At the time of the primary analysis, the subgroup analyses showed that OS was better in the GS group than in the gemcitabine group among patients with PS 1 as well as in those with locally advanced disease (Ueno et al. 2013). In addition, a similar trend was seen with the results of analyses in this long-term follow-up. The hazard ratios of OS in patients with a PS of 1 was 0.74 (95% CI 0.56–0.98) and in those with locally advanced disease was 0.73 (95% CI 0.51–1.04). OS did not differ among the three treatment groups in patients with PS 0.

In the GS group, the mOS was similar in patients with PS 0 and those with PS 1 (PS 0, 10.5 months; PS 1, 9.6 months). In contrast, PS 1 was associated with a trend toward a shorter mOS in both the gemcitabine group (PS 0, 9.8 months; PS 1, 6.2 months) and S-1 group (PS 0, 10.9 months; PS 1, 6.3 months). In the gemcitabine group and S-1 group, the proportions of patients who received both drugs (gemcitabine and S-1) in the first-line and second-line therapies were lower in patients with PS 1 than in those with PS 0. In addition, the HR for PFS of PS 1 in the GS group compared with that in the gemcitabine group was very small (HR 0.52; 97.5% CI 0.37–0.73).

These findings suggest that the longer PFS obtained by GS may have had a substantial impact on OS, especially in the subset of patients with PS 1, because the patients with PS 1 in the gemcitabine group and S-1 group could not adequately receive effective available drugs as a second-line treatment for pancreatic cancer. Consequently, survival was unable to be adequately prolonged without using both active drugs.

Better PFS were observed in the GS group for both patients with metastases and locally advanced disease; the PFS in patients with metastases was 3.0 and 5.4 months in the gemcitabine and GS groups, respectively, and PFS in patients with locally advanced cancer was 6.2 and 10.7 months in the gemcitabine and GS groups, respectively. On the other hand, the OS was almost the same for the gemcitabine and GS groups in patients with metastases (HR 0.96), although a longer OS was observed in the GS group compared with the gemcitabine group in patients with locally advanced disease (HR 0.73). The reason for the differences in trends in OS observed between patients with locally advanced cancer and metastases was unclear. However, tumor shrinkage was greater in the GS group (29.3%) than the gemcitabine group (13.3%), which might have contributed to the prolonged survival in patients with locally advanced disease (HR = 0.73) by the mechanism of controlling micrometastasis. Tumor shrinkage was also slightly greater in S-1 alone group (21.0%) than gemcitabine alone group (13.3%), while OS in patients with locally advanced disease patients also showed a preferable trend in the S-1 group (HR = 0.81, Fig. 2). On the other hand, since tumor burden at metastatic focuses had increased in patients with metastatic disease, the effect of reduced micrometastasis may not have given as much impact as OS prolongation in the GS group.

Although our results were obtained from a single clinical trial, the median follow-up of the survivors (29.8 months) and the number of events related to OS (795 events; 95.6%) were properly mature. Therefore, our results for survival are considered to be robust. We believe that the survival rates obtained in our study can serve as reference for future studies evaluating the outcomes of patients with pancreatic cancer who receive chemotherapy in East Asia (Supplemental Table 1).

In recent clinical studies of pancreatic cancer, patients with locally advanced disease have been evaluated separately from those with metastatic disease. In our study, patients with both metastatic and locally advanced disease were enrolled. Our subgroup analyses revealed a trend toward better outcomes in patients with locally advanced disease than in those with metastatic disease, in all treatment groups (Supplemental Table 1). Because the present study evaluated the same treatment regimens in patients with locally advanced disease and those with metastatic disease, our results might be useful as reference data for the future development of treatments for pancreatic cancer.

Our follow-up survey reconfirmed the non-inferiority of S-1 to gemcitabine and showed that S-1 can be used as a first-line treatment for both locally advanced and metastatic pancreatic cancer. The detailed analysis of data for long-term survival in our study might contribute toward the development of new treatments for pancreatic cancer.

## Electronic supplementary material

Below is the link to the electronic supplementary material.

Study flow chart (CONSORT diagram) (TIF 387 KB)

Percentage changes from baseline to nadir of the sum of the longest diameter of target lesions. (a) Gemcitabine vs. GS in pancreatic lesions. (b) Gemcitabine vs. S-1 in pancreatic lesions. (c) Gemcitabine vs. GS in metastatic lesions. (d) Gemcitabine vs. S-1 in metastatic lesions. Abbreviations: GEM, gemcitabine; GS, gemcitabine plus S-1 (TIF 836 KB)

Supplementary material 3 (DOCX 19 KB)

Supplementary material 4 (DOCX 16 KB)
