# Supplementary figures and images for: Updated results from GEST study: a randomized, three-arm phase III study for advanced pancreatic cancer
Source: J Cancer Res Clin Oncol. 2017 Feb 16;143(6):1053–9. doi: 10.1007/s00432-017-2349-y (PMC5427167; doi:10.1007/s00432-017-2349-y)

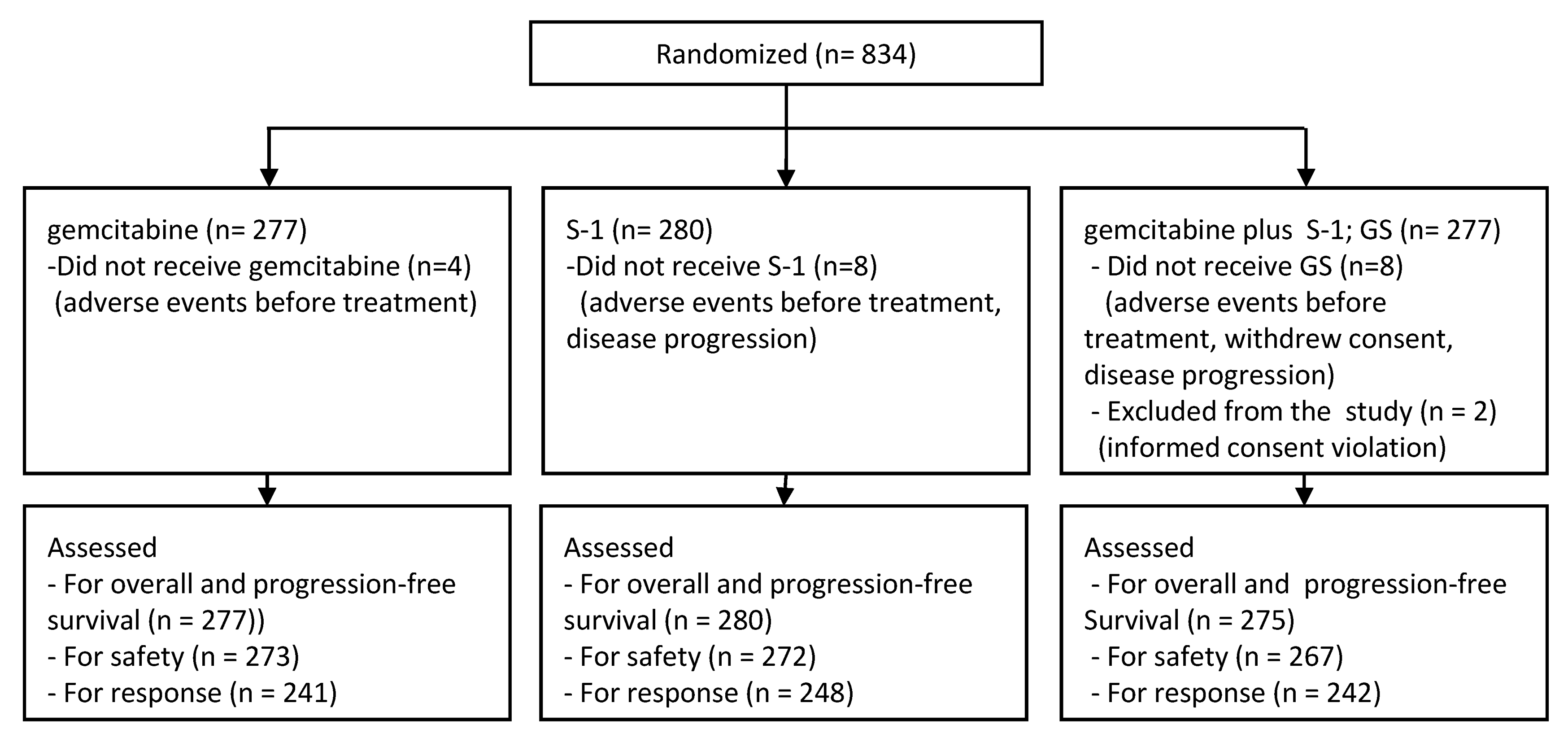

Supplement: Supplementary file 1 — Study flow chart (CONSORT diagram) (TIF 387 KB) [file 432_2017_2349_MOESM1_ESM.tif]

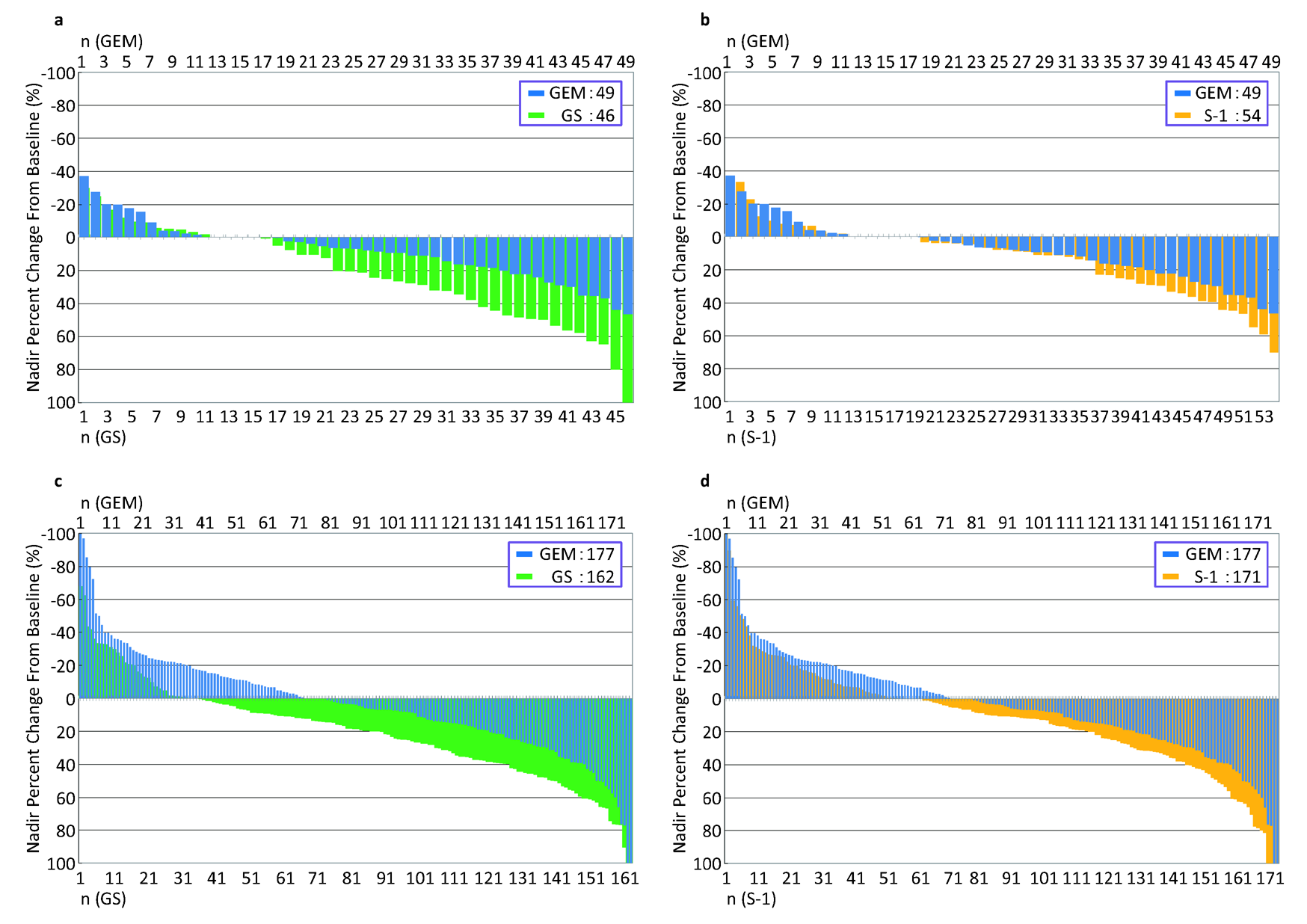

Supplement: Supplementary file 2 — Percentage changes from baseline to nadir of the sum of the longest diameter of target lesions. (a) Gemcitabine vs. GS in pancreatic lesions. (b) Gemcitabine vs. S-1 in pancreatic lesions. (c) Gemcitabine vs. GS in metastatic lesions. (d) Gemcitabine vs. S-1 in metastatic lesions. Abbreviations: GEM, gemcitabine; GS, gemcitabine plus S-1 (TIF 836 KB) [file 432_2017_2349_MOESM2_ESM.tif]
