# Supplementary material for: Updated results from GEST study: a randomized, three-arm phase III study for advanced pancreatic cancer
Source: J Cancer Res Clin Oncol. 2017 Feb 16;143(6):1053–9. doi: 10.1007/s00432-017-2349-y (PMC5427167; doi:10.1007/s00432-017-2349-y)
Supplement: Supplementary file 3 — Supplementary material 3 (DOCX 19 KB) [file 432_2017_2349_MOESM3_ESM.docx]

Supplemental Table 1

|  | Treatment | *n* | *OS* | Median survival time | |  | 1y-survival rate | |  | 2y-survival rate | |  | 3y-survival rate | |
| --- | --- | --- | --- | --- | --- | --- | --- | --- | --- | --- | --- | --- | --- | --- |
|  | group |  | *Event* | months | 95% CI |  | % | 95% CI |  | % | 95% CI |  | % | 95% CI |
| All | GEM | 277 | 267 | 8.8 | 8.0-9.7 |  | 35.0 | 29.4-40.6 |  | 9.4 | 6.3-13.2 |  | 3.4 | 1.5-6.4 |
|  | S-1 | 280 | 264 | 9.7 | 7.6-10.8 |  | 38.4 | 32.6-44.1 |  | 10.9 | 7.5-15.0 |  | 3.6 | 1.7-6.6 |
|  | GS | 275 | 264 | 9.9 | 9.0-11.2 |  | 40.4 | 34.5-46.1 |  | 11.6 | 8.2-15.7 |  | 4.1 | 2.1-7.1 |
| Metastatic | GEM | 211 | 203 | 8.3 | 7.0-8.9 |  | 29.4 | 23.4-35.6 |  | 7.1 | 4.2-11.1 |  | 3.7 | 1.7-7.2 |
|  | S-1 | 212 | 201 | 7.4 | 6.6-9.6 |  | 32.4 | 26.1-38.8 |  | 7.6 | 4.4-11.8 |  | 2.6 | 0.9-5.7 |
|  | GS | 207 | 202 | 9.3 | 7.7-10.1 |  | 32.9 | 26.6-39.3 |  | 8.2 | 5.0-12.5 |  | 2.7 | 1.0-6.0 |
| Locally advanced | GEM | 66 | 64 | 12.7 | 9.7-14.9 |  | 53.0 | 40.4-64.2 |  | 16.7 | 8.9-26.6 |  | 2.5 | 0.2-10.5 |
|  | S-1 | 68 | 63 | 13.8 | 11.0-16.9 |  | 56.8 | 44.2-67.7 |  | 20.9 | 12.2-31.3 |  | 6.3 | 1.8-15.0 |
|  | GS | 68 | 62 | 15.9 | 13.0-19.7 |  | 63.2 | 50.6-73.4 |  | 22.1 | 13.1-35.5 |  | 8.2 | 3.1-16.7 |
| Japan | GEM | 256 | 249 | 8.9 | 8.3-9.7 |  | 35.9 | 30.1-41.8 |  | 8.2 | 5.3-12.0 |  | 3.1 | 1.3-5.9 |
|  | S-1 | 257 | 245 | 9.7 | 7.7-10.9 |  | 39.2 | 33.2-45.2 |  | 10.5 | 7.0-14.7 |  | 3.5 | 1.6-6.5 |
|  | GS | 253 | 243 | 9.7 | 8.9-10.9 |  | 39.9 | 33.9-45.9 |  | 11.9 | 8.2-16.2 |  | 4.3 | 2.2-7.5 |
| Taiwan | GEM | 21 | 18 | 5.3 | 4.2-10.8 |  | 23.8 | 8.7-43.1 |  | 23.8 | 8.7-43.1 |  | - | - |
|  | S-1 | 23 | 19 | 6.4 | 4.8-11.4 |  | 29.0 | 12.2-48.2 |  | 17.4 | 4.7-36.6 |  | - | - |
|  | GS | 22 | 21 | 11.2 | 8.0-17.1 |  | 45.5 | 24.4-64.3 |  | 9.1 | 1.6-25.1 |  | - | - |
| Abbreviations: CI; confidence interval, GEM; gemcitabine, GS; gemcitabine plus S-1. | | | | | | | | | | | | | | |
