# Supplementary material for: Updated results from GEST study: a randomized, three-arm phase III study for advanced pancreatic cancer
Source: J Cancer Res Clin Oncol. 2017 Feb 16;143(6):1053–9. doi: 10.1007/s00432-017-2349-y (PMC5427167; doi:10.1007/s00432-017-2349-y)
Supplement: Supplementary file 4 — Supplementary material 4 (DOCX 16 KB) [file 432_2017_2349_MOESM4_ESM.docx]

Supplemental Table 2

|  | Pancreatic lesions | | | Metastatic lesions | | |
| --- | --- | --- | --- | --- | --- | --- |
|  | GEM | S-1 | GS | GEM | S-1 | GS |
| Variable | n=49 | n=54 | n=46 | n=177 | n=171 | n=162 |
| Baseline, mm |  |  |  |  |  |  |
| Median | 37.5 | 40.0 | 40.0 | 58.0 | 62.0 | 66.0 |
| (range) | (22.0-110.0) | (12.0-110.0) | (19.0-97.0) | (10.0-271.0) | (10.0-326.0) | (10.0-268.0) |
| Change at Nadir, % |  |  |  |  |  |  |
| Median | -7.0 | -7.9 | -20.9 | -3.6 | -10.4 | -18.8 |
| (range) | (-46.7-37.3) | (-70.2-34.4) | (-100.0-30.0) | (-100.0-106.6) | (-100.0-111.3) | (-100.0-68.1) |
| Abbreviations: CI; confidence interval, GEM; gemcitabine, GS; gemcitabine plus S-1. | | | | | | |
